# Successful Treatment of Pulmonary Arterial Hypertension in Systemic Sclerosis with Anticentriole Antibody

**DOI:** 10.1155/2020/1926908

**Published:** 2020-02-25

**Authors:** Yusho Ishii, Hiroshi Fujii, Koichiro Sugimura, Tsuyoshi Shirai, Yosuke Hoshi, Yoko Fujita, Yuko Shirota, Tomonori Ishii, Hiroaki Shimokawa, Hideo Harigae

**Affiliations:** ^1^Department of Hematology and Rheumatology, Tohoku University Graduate School of Medicine, Aoba-ku, Sendai, Japan; ^2^Department of Cardiovascular Medicine, Tohoku University Graduate School of Medicine, Aoba-ku, Sendai, Japan; ^3^Clinical Research, Innovation and Education Center, Tohoku University Hospital, Sendai, Miyagi, Japan

## Abstract

Systemic sclerosis (SSc) is characterized by skin sclerosis and multiple organ damages which may cause mortality and is usually accompanied with several specific autoantibodies, each of which is associated with characteristic complications. Among them, anticentriole antibody is recently reported to be highly associated with SSc-associated pulmonary arterial hypertension (SSc-PAH). In general, several vasodilators are used as therapeutic drugs for SSc-PAH, whereas immunosuppressive therapies are not. Here, we report the case of a 62-year-old female with anticentriole antibody-positive SSc-PAH treated with immunosuppressants and vasodilators. She presented with two-year exertional dyspnea and was diagnosed with PAH and SSc owing to the centriole staining pattern and other symptoms without digital sclerosis. Oral vasodilators were initially administered but were not sufficiently effective on dyspnea. Immunosuppressants such as prednisolone and cyclophosphamide were started. Both of them improved mean pulmonary arterial pressure and 6-minute walk distance, and the anticentriole antibody also disappeared. In this case, SSc-PAH with anticentriole antibody was properly diagnosed and immunosuppressants and vasodilators improved the hemodynamics of PAH with anticentriole antibody and stably maintained it and, in addition, reduced the titer of anticentriole antibody. This indicates that anticentriole antibody might represent a good responsive group to therapies among subgroups of patients with SSc-PAH.

## 1. Introduction

Systemic sclerosis (SSc) is a systemic autoimmune disease and presents with vasculopathy, inflammation, and fibrosis of the skin and internal organs [1, 2]. SSc also presents with heterogeneous organ damages such as pulmonary arterial hypertension (PAH) and interstitial pneumoniae (IP), gastrointestinal dysfunction, cardiac dysfunction, and skin disorder [1, 2]. PAH is one of the serious complications that induce high mortality. Generally, the recommended treatments for SSc-PAH comprise vasodilators, including phosphodiesterase-5 inhibitor (PDE5i), endothelin receptor antagonist, prostacyclin analogs, and soluble guanylate cyclase stimulator [3], which repress the rapid progression of SSc-PAH [4]. Although immunosuppressive therapies are not generally effective for SSc-PAH compared with other connective tissue diseases associated with PAH [2, 5], clinical trials for rituximab, tocilizumab, and dimethyl fumarate for SSc-PAH are underway and are effective in some cases of SSc-PAH [6–9]. Some case reports have indicated that a subpopulation of patients with SSc-PAH is responsive to immunosuppressive therapy [10, 11]; however, it still remains unclear what kind of clinical features or biomarkers are useful to identify SSc-PAH patients on whom immunosuppressive therapies are effective.

Patients with SSc show various types of autoantibodies, and each autoantibody is associated with characteristic clinical phenotypes such as anti-topoisomerase I (diffuse sclerosis, IP, digital ulcer (DU), and severe heart disease), anti-centromere (limited sclerosis, DU, calcinosis, and PAH), anti-RNA polymerase III (diffuse sclerosis and renal crisis), anti-U3 RNP (diffuse sclerosis, PAH, IP, severe heart disease, myositis, and overlap syndrome), anti-Th/To (limited sclerosis, PAH, and IP), anti-PM-Scl (limited sclerosis and SSc-myositis overlap syndrome), and anti-Ku antibodies (overlap syndrome and myositis) [12–14]. In addition, the anticentriole antibody is recently reported to be highly associated with PAH in patients with SSc [15]. Here, we present the case of a patient with SSc-PAH with anticentriole antibody who was successfully treated with vasodilators and immunosuppressive therapies. The presence of the anticentriole antibody is rare; however, it may be a useful biomarker that affects diagnostic and therapeutic strategies for SSc-PAH.

## 2. Case Presentation

**Figure 1:**
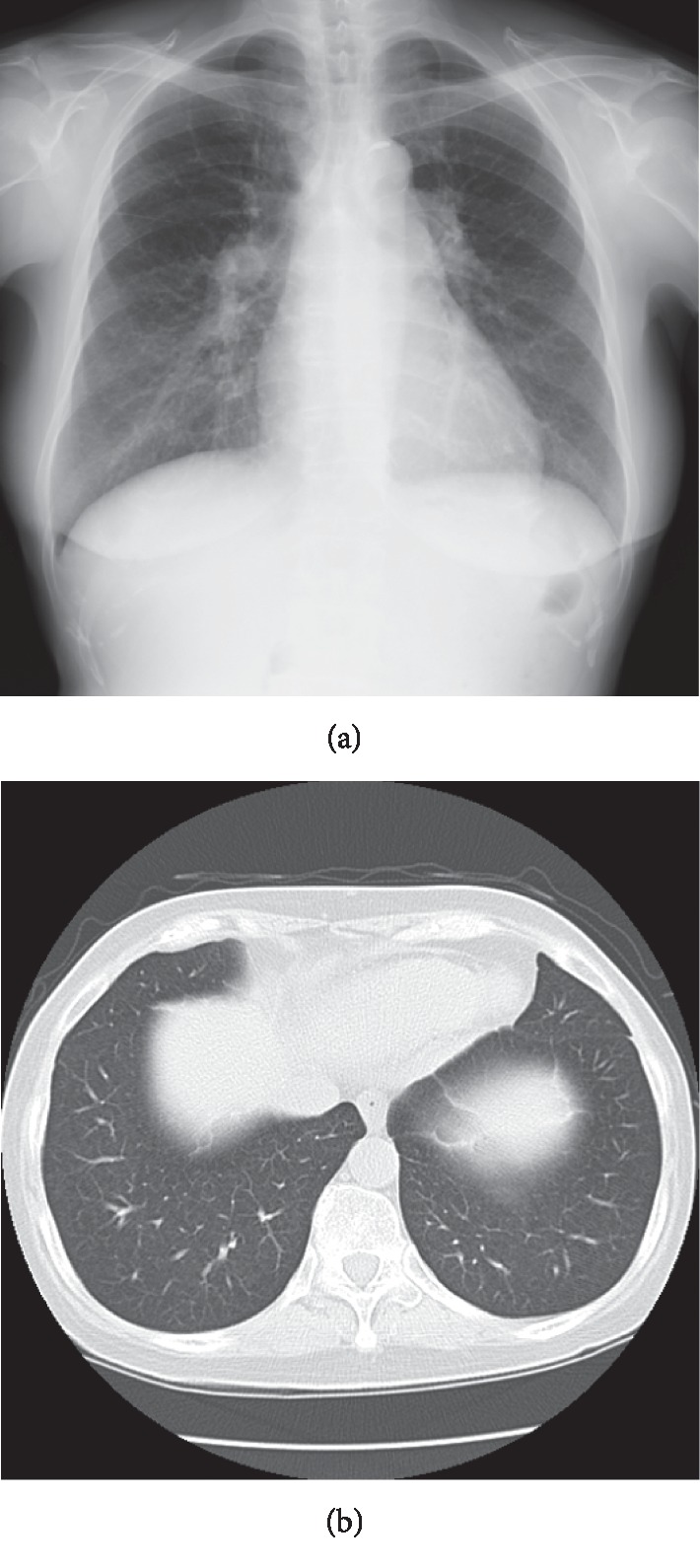
Chest X-ray and computed tomography examination performed.

**Figure 2:**
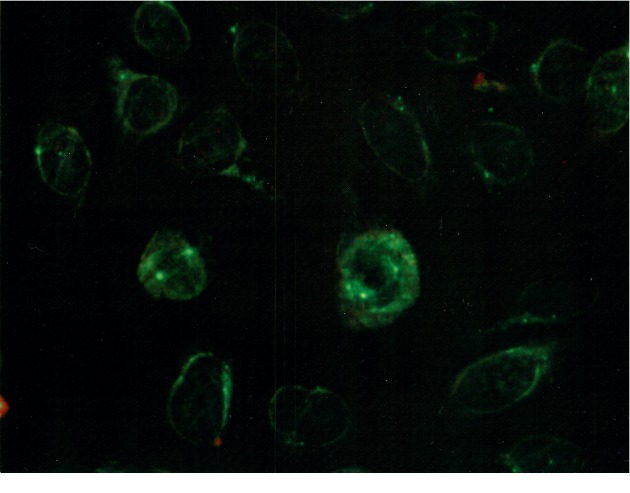
Anticentriole antibody in immunofluorescence examination (1 : 640).

**Figure 3:**
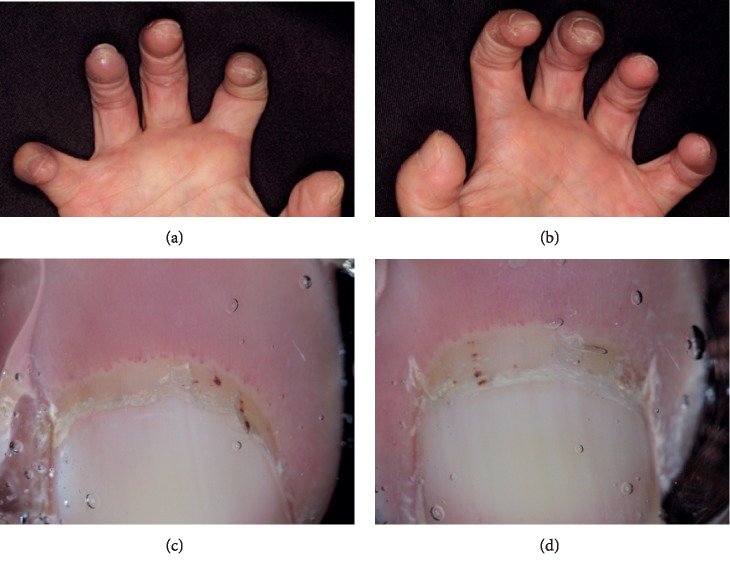
Scar of the digital ulcer found and nailfold bleeding.

**Figure 4:**
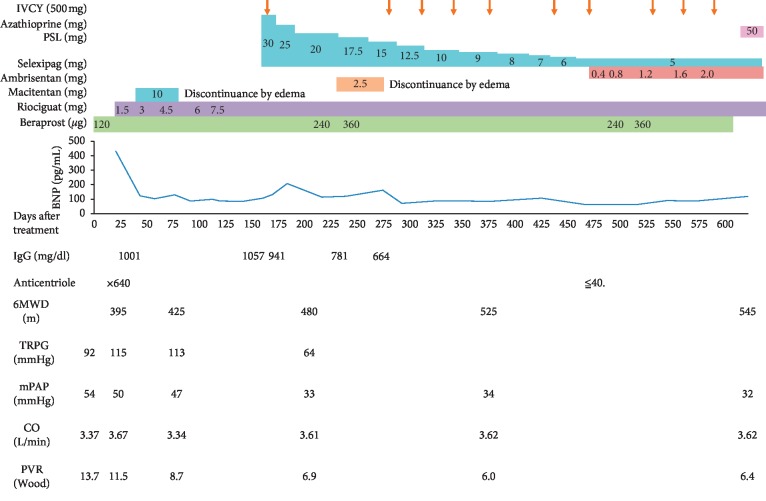
Clinical course.

A 62-year-old female presented with two-year exertional dyspnea. Her dyspnea progressively worsened and severe pitting edema of the lower extremities appeared, so she visited her previous doctor and took an electrocardiography which revealed right heart overload. Blood examination showed that the value of brain natriuretic peptide (BNP) was 537 pg/ml. Echocardiography revealed that the ejection fraction was 78.7%. Tricuspid regurgitation pressure gradient (TRPG) was elevated to 92 mmHg, and the right atrium, right ventricle, and inferior vena cava were enlarged. The cardiac catheter test revealed that the mean pulmonary arterial pressure (mPAP), pulmonary capillary wedge pressure, and pulmonary vascular resistance (PVR) levels were 54 mmHg, 8 mmHg, and 13.7 Wood, respectively. The pulmonary function test revealed the percent vital capacity (%VC), FEV_1.0_%, and percent diffusing capacity of the lung for carbon monoxide (%DLCO) to be 117%, 82%, and 55%, respectively, thus suggesting the involvement of the lungs with impaired diffusion but not with restrictive and obstructive pulmonary disorder. Her chest X-ray and computer tomography did not reveal any pulmonary complications (Figure 1). She was diagnosed with group I PAH according to the Nice classification of PH. Serological analysis revealed antinuclear antibody (ANA) to be negative; however, the centriole pattern of staining was observed with high titers (1 : 640) using indirect immunofluorescence (Figure 2). Then, she was introduced to our hospital. The patient also manifested DUs, nailfold bleeding, telangiectasia (Figure 3), and Raynaud's phenomenon but no skin sclerosis when she visited us. She has never noticed her digital sclerosis. Based on the 2013 classification criteria for SSc by the American College of Rheumatology/European League against Rheumatism, the patient was subsequently diagnosed with SSc [16] (Table 1). She was initially treated with oral vasodilators and diuretics. Beraprost (120 *μ*g/day) and riociguat (1.5 mg/day) as vasodilators were prescribed and azosemide (60 mg/day), spironolactone (25 mg/day), and tolvaptan (3.75 mg/day) as diuretics were also prescribed in order. As edema of the lower limbs worsened immediately after macitentan was started, its use was discontinued. Dyspnea was not completely improved with beraprost and riociguat. Although we considered continuous intravenous infusion of epoprostenol as an additional therapy, the patient refused because of issues concerning aesthetics and quality of life. Therefore, we discussed with cardiologists and suggested the use of immunosuppressive therapy, which might be effective owing to the positivity of the anticentriole antibody, a unique immunological abnormality strongly associated with SSc-PAH. Moderate doses of prednisolone (PSL; 30 mg/day) and intravenous cyclophosphamide (IVCY; 500 mg every four weeks) were initiated. After two months, these immunosuppressive therapies and vasodilators improved mPAP from 47 to 33 mmHg, 6-minute walk distance (6MWD) from 418 to 480 m, and PVR from 8.7 to 6.0 Wood. Interestingly, the anticentriole antibody also disappeared and was not detected 16 months with a slight decrease of immunoglobulin G (IgG) after the treatment. After 10 times IVCY, azathioprine (50 mg/day) was started, and PSL was gradually decreased and maintained at 5 mg/day. To achieve further improvement, a new vasodilator selexipag was added and increased up to 2 mg/day in addition to riociguat (7.5 mg/day) and beraprost (360 mg/day). PAH has been well controlled using these treatments (Figure 4).

## 3. Discussion

PAH is one of the most severe complications of SSc. Approximately, 10% of all patients with SSc experience PAH [17]. The associated PAHs in SSc, systemic lupus erythematosus (SLE), mixed connective tissue disease (MCTD), and rheumatoid arthritis (RA) are 68%, 19%, 9%, and 5%, respectively [18]. Primary Sjögren syndrome (SS) has a very low prevalence of PAH [19]. SSc-PAH has a worse prognosis compared with other CTD-PAH, partially because the PAH of SLE, MCTD, SS, and RA were improved with the administration of immunosuppressive drugs (cyclophosphamide, prednisolone, azathioprine, and mycophenolate mofetil) [18–23]. Therefore, except for cases of progressive skin and pulmonary fibrosis, we generally do not use immunosuppressive therapies for patients with SSc [24].

Centrosome plays an important role in dividing cells with the mitotic spindle during cell cycle. It is usually located near the nucleus and moves to the bipolar of the cells after its replication during cell division. This means that the centrioles are one of the components of the centrosome located in the cytoplasm. We can detect the anticentriole antibody by the examination of antinuclear antibodies; however, it is not the antinuclear antibody but the anticytoplasmic antibody [25]. Centrosome is composed of centrioles and proteins termed the pericentriolar material (PCM) and is responsible for microtubule nucleation and anchoring [25]. Some centrosomal proteins have autoreactivities such as pericentriolar material 1 (PCM-1), pericentrin, ninein, and Cep250. Antibodies for those proteins were identified from autoimmune disease patients and postinfectious patients [26]. There is high prevalence of centrosome antibodies in SSc among autoimmune diseases [27]. Anticentriole antibody is one of anticentrosome antibodies. Connolly et al. first identified the antibody for centriole in nonimmune rabbit sera in 1978 [28]. Since 1980, several reports have been published regarding the anticentriole antibody. In these reports, the anticentriole antibody was indicated to be related to SSc [29–33]. Recently, Hamaguchi reported that 4 of 5 patients with the anticentriole antibody in SSc develop Raynaud's phenomenon and PAH [15]. Clinically, this autoantibody is not well recognized or elucidated as an SSc-specific antibody. Using previous PubMed reports, we investigated the characteristics of patients with positive anticentriole antibody. Although some data were unavailable, we found 16 cases of patients with anticentriole antibody between 1982 and 2015. Based on those reports, 11 of 11 (100%) patients were female. Reynaud phenomenon, DU, and PAH were observed at high rates in 100% (14 of 14), 86% (6 of 7), and 71% (5 of 7) of cases, respectively. The anticentriole antibody was reported to be associated with SSc and 4 of 15 patients (27%) did not show any cutaneous sclerosis (Table 2) [15, 30–32, 34–37]. This ratio of patients without skin sclerosis (27%) was higher than that of all patients with SSc, because only 5%–9% of SSc were classified as SSc sine sclerosis [38–40]. In addition, the anticentriole antibody is not detected as ANA; hence, such patients without clear skin sclerosis may be missed and diagnosed with “idiopathic PAH.” When an ANA-negative patient presents with DUs, Raynaud phenomenon, and/or PAH, the original image of immunofluorescence of ANA staining should be reconsidered for the presence of anticentriole antibody.

To the best of our knowledge, this is the first report of an SSc-PAH patient with anticentriole antibody, who was successfully treated. In this case, the hemodynamics of PAH were further improved and stably maintained after the induction of immunosuppressive therapies. The positivity of the anticentriole antibody in SSc is extremely highly associated with PAH among other SSc-associated autoantibodies [15]. Immunosuppressive therapies decreased the titer of the anticentriole antibody. These results indicate that the pathogenesis of SSc-PAH with the anticentriole antibody may be immune-mediated. Thus, the anticentriole antibody could be a useful biomarker to identify a subpopulation of patients with SSc-PAH.

In mitotic phase, centrioles at poles of mitotic spindles were stained like 2 dots.

Anticentriole antibody was detected in 1 : 640 by a commercial examination. The staining disappeared completely after the treatment (no data shown).

## Figures and Tables

**Table 1:** Peripheral blood examination inday 20 after treatment.

Blood test (day 20)	

*Urinalysis*	
Specific gravity	1.021
pH	6
Protein	+
Blood	–
Nitrite	–
WBC	–
Bacteria	–
*CBC*	
WBC	5,000 mL
Neutrophil	3,000 mL
Eosinophil	0 mL
Basophil	0 mL
Lymphocyte	1,800 mL
Monocyte	200 mL
RBC	4.77 × 10^6^ mL
Hb	13.5 g/dL
MCV	89.5 fl
Plt	212 × 10^3^ mL

*Coagulation*	
PT	18.7 sec
PT-INR	2.34
APTT	52.9 sec
D-dimer	0.6 mg/mL
*Infection*	
T-SPOT	(−)
HBs Ab	(−)
HBs Ag	(−)
HCV Ab	(−)

*Biochemistry*	
T-bil	1.6 mg/dL
D-bil	0.1 mg/dL
ALP	72 U/L
*γ* − GTP	33 U/L
AST	35 U/L
ALT	34 U/L
LDH	297 U/L
BUN	10 mg/dL
Cre	0.55 mg/dL
UA	4.4 mg/dL
TP	6.4 g/dL
Alb	4 g/dL
Pre-Alb	15 mg/dL
Na	142 mEq/L
K	3.7 mEq/L
Cl	107 mEq/L
Ca	8.4 mg/dL
IP	3.6 mg/dL
TG	101 mg/dL
TC	122 mg/dL
LDL-C	69 mg/dL
CPK	112 U/L
CK-MB	21 U/L
Glucose	98 mg/dL
Hb-A1c (NGSP)	6.2%
CRP	0.07 mg/dL
Hp	≦3.0 mg/dL
Ferritin	69.8 ng/mL

*Endocrine*	
FT4	1.17 ng/dL
TSH	6.33 mgIU/mL
FT3	2.69 pg/mL
BNP	432.1 pg/mL

*Immune system*	
IgG	1001 mg/dL
IgA	228 mg/dL
IgM	94 mg/dL
ANA	≦40
C3	90 mg/dL
C4	21.8 mg/dL
CH50	46.9 U/mL
Anti-dsDNA Ab	≦10I U/mL
Anti-RNP Ab	≦5.0 U/mL
Anti-Sm Ab	≦5.0 U/mL
Anti-SS-A/Ro Ab	≦5.0 U/mL
Anti-SS-B/La Ab	≦5.0 U/mL
Anti-Scl 70 Ab	≦5.0 U/mL
Anti-centromere Ab	≦5.0 U/mL
Anti-CL.*β*2GPI Ab	≦1.3 U/mL
Anti-CALG Ab	≦1.0 U/mL
LAC	≦1.0 U/mL
PR3-ANCA	≦1.0I U/mL
MPO-ANCA	≦1.0 U/mL
RF	≦5.0 U/mL
Anti-CCP Ab	≦0.5 U/mL
Anti-ARS Ab	10 U/mL
Anti-RNA polymerase III Ab	15.2

**Table 2:** Data about anticentriole antibody positive patients from previous reports Ref [15, 30–32, 34–37].

The feature of anticentriole antibody positive cases from previous reports				
Total 16 cases *n* (%)	Positive	Negative	Not described	*P*/*P* + *N* (%)
Sex (female)	11 (69)	0 (0)	5 (31)	100
Raynaud's phenomenon	14 (88)	0 (0)	2 (12)	100
Digital sclerosis	9 (56)	4 (25)	3 (19)	69
Systemic sclerosis	4 (25)	9 (56)	3 (19)	31
Digital ulcer	6 (38)	1 (6)	9 (56)	86
Interstitial pneumonia	4 (25)	6 (38)	6 (38)	40
Reflux esophagitis	5 (31)	6 (38)	5 (31)	45
Telangiectasia	5 (31)	5 (31)	6 (38)	50
Renal crisis	0 (0)	8 (50)	8 (50)	0
Pulmonary hypertension	5 (31)	2 (12)	9 (56)	71

## Abbreviations

IVCY: Intravenous cyclophosphamide
PSL: Prednisolone
BNP: Brain natriuretic peptide
IgG: Immunoglobulin G
Ab: Antibody
6MWD: 6-minute walk distance
TRPG: Transtricuspid pressure gradient
mPAP: Mean pulmonary arterial pressure
CO: Cardiac output
PVR: Pulmonary vascular resistance.
